# Early morbidity and dose–volume effects in definitive radiochemotherapy for locally advanced cervical cancer: a prospective cohort study covering modern treatment techniques

**DOI:** 10.1007/s00066-021-01781-6

**Published:** 2021-04-30

**Authors:** Yvette Seppenwoolde, Katarina Majercakova, Martin Buschmann, Elke Dörr, Alina E. Sturdza, Maximilian P. Schmid, Richard Pötter, Dietmar Georg

**Affiliations:** 1 Department of Radiation Oncology, Comprehensive Cancer Center, Medical University of Vienna/AKH Wien, Währinger Gürtel 18–20, 1090 Vienna, Austria; 2 Christian Doppler Laboratory for Medical Radiation Research for Radiation Oncology, Medical University of Vienna, Vienna, Austria; 3 Department of Radiation Oncology, Hospital de la Santa Creu i Sant Pau, Barcelona, Spain; 4 Department of Radiation Oncology, Erasmus MC Cancer Institute, Rotterdam, The Netherlands

**Keywords:** Image-guided adaptive radiotherapy, Radiotherapy, Brachytherapy, Organs-at-risk toxicity, Quality of life

## Abstract

**Purpose:**

Predicting morbidity for patients with locally advanced cervix cancer after external beam radiotherapy (EBRT) based on dose–volume parameters remains an unresolved issue in definitive radiochemotherapy. The aim of this prospective study was to correlate patient characteristics and dose–volume parameters to various early morbidity endpoints for different EBRT techniques, including volumetric modulated arc therapy (VMAT) and adaptive radiotherapy (ART).

**Methods and materials:**

The study population consisted of 48 patients diagnosed with locally advanced cervix cancer, treated with definitive radiochemotherapy including image-guided adaptive brachytherapy (IGABT). Multiple questionnaires (CTCAE 4.03, QLQ-C30 and EORTC QLQ-CX24) were assessed prospectively for patients treated with different EBRT techniques, including online adaptive VMAT. Contouring and treatment planning was based on the EMBRACE protocols. Acute toxicity, classified as general, gastrointestinal (GI) or genitourinary (GU) and their corresponding dose–volume histograms (DVHs) were first correlated by applying least absolute shrinkage and selection operator (LASSO) and subsequently evaluated by multiple logistic binomial regression.

**Results:**

The treated EBRT volumes varied for the different techniques with ~2500 cm^3^ for 3D conformal radiotherapy (3D-CRT), ~2000 cm^3^ for EMBRACE‑I VMAT, and ~1800 cm^3^ for EMBRACE-II VMAT and ART. In general, a worsening of symptoms during the first 5 treatment weeks and recovery afterwards was observed. Dose–volume parameters significantly correlating with stool urgency, rectal and urinary incontinence were as follows: bowel V_40Gy_ < 250 cm^3^, rectum V_40Gy_ < 80% and bladder V_40Gy_ < 80–90%.

**Conclusion:**

This prospective study demonstrated the impact of EBRT treatment techniques in combination with chemotherapy on early morbidity. Dose–volume effects for dysuria, urinary incontinence, stool urgency, diarrhea, rectal bleeding, rectal incontinence and weight loss were found.

## Introduction

Radiation oncology plays a major role in the treatment of locally advanced cervical cancer. Current standard of care is concurrent radiochemotherapy combining external beam radiotherapy (EBRT) with cisplatin followed by a brachytherapy (BT) boost, preferably by image-guided adaptive brachytherapy (IGABT; [1]).

Advanced EBRT techniques, especially those based on intensity modulation, enabled improved organ-at-risk (OAR) sparing and consequently decreased the incidence of severe toxicity (G3, G4) [2, 3]. Intensity modulated radiotherapy (IMRT) and volumetric modulated arc therapy (VMAT) with small margins are challenging in cervical cancer, because of large intra- and interfraction motion [4–7]. Therefore, adaptive radiotherapy (ART) that adjusts treatment plans according to organ movements to further reduce irradiated volumes has become a main research interest [8–15].

Despite these advancements, 60–70% of patients still experience early lower grade (G1–2) side effects, of which 30% develop into late morbidity [16, 17]. Several publications concluded that especially gastrointestinal (GI), genitourinary (GU) and vaginal/sexual problems have an impact on quality of life (QoL) [8, 18, 19]. Furthermore, observational studies demonstrated that radiochemotherapy leads to more physical, psychological and sexual sequelae [18, 20–22], especially in premenopausal patients. However, none of those studies correlated side effects with dose distributions.

Unraveling the EBRT effects from brachytherapy and chemotherapy effects is challenging. At the time of BT, the full EBRT dose might not have yet been delivered and is mostly in the lower range of the tolerance dose (TD_50_) for rectum and bladder [23]. On the contrary, and based on the authors’ experience, the recommended dose–volume parameter for small bowel (V_45Gy_ < 195 cm^3^) is exceeded for most cervix patients. For small bowel tolerance, no difference in the incidence of small bowel toxicity was found for doses from 5 to 40 Gy [24], while another study concluded that V_16Gy_ should be <290 cm^3^ for patients without prior abdominal surgery to prevent >G2 acute diarrhea [25]. For bladder, grade 3 late toxicity was found to occur for doses >50 Gy [26] and the TD_50_ of patient reported symptoms is often >85 Gy [27]. Late rectal injury was found to be rare in current dose ranges [28].

The aim of this mono-institutional observational study was to assess early morbidity as a function of EBRT dose distributions for different techniques, varying from 3D-CRT to VMAT-ART, the latter being based on a bladder filling/uterus motion model. Early side effects were assessed using multiple questionnaires (patient and physician reported). Dose–volume parameters as well as patient demographics were correlated with early morbidity at different time points with the aim to determine dose–effect relations and predictive factors.

## Materials and methods

### Patient cohorts and treatment

The inclusion criteria were: age ≥18 years, histologically proven locally advanced cervix cancer FIGO Ib–IVb (para-aortic lymph node metastasis), no previous radio- or chemotherapy, patients suitable for definitive treatment, no other diagnosis of tumor and patients capable to treatment and study compliance.

**Fig. 1:**
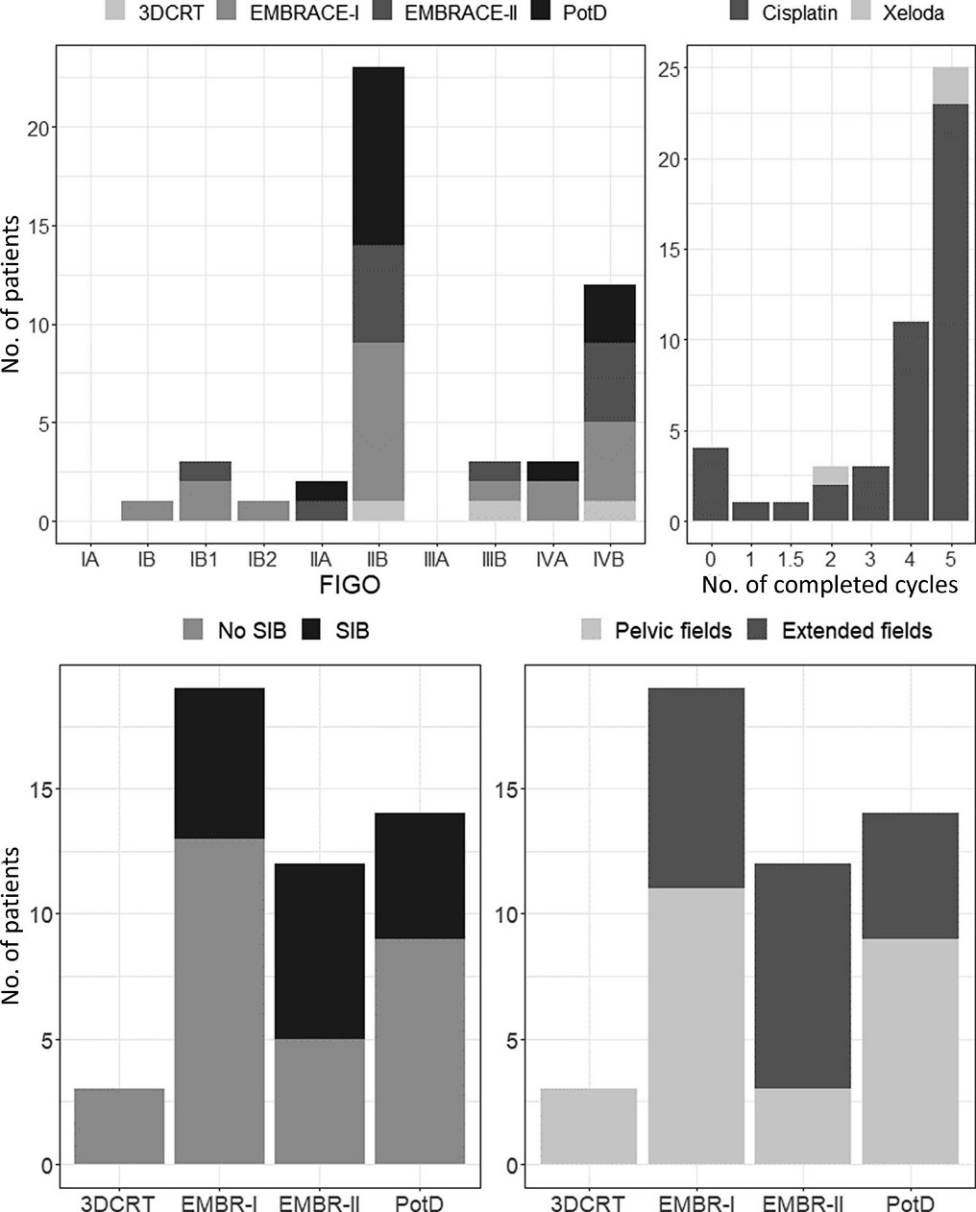
Distribution of patients over the FIGO stages, number of completed chemotherapy cycles, simultaneous integrated boost (SIB), extended fields (PAN) and irradiation technique. *Embrace-I//II *patients were delineated and planned according to the respective study protocols, but not all were included in those studies. *PotD* plan of the day, *3DCRT 3D* conformal radiotherapy

All 48 included patients were treated with definitive radiochemotherapy including consecutive MRI-guided IGABT. A total of 44 patients (92%) received chemotherapy. Patient-specific parameters (PsP) like smoking, alcohol, chemotherapy (both regimen and the number of completed cycles), the use of para-aortic nodal fields (PAN) and age were collected as well. The patients’ characteristics of this ethics-committee-approved study are summarized in Fig. 1.

Target and organ-at-risk (OAR) delineation as well as treatment planning were based either on EMBRACE‑I and later EMBRACE-II guidelines [29], though not all patients were participating in those studies. The bowel bag was recontoured as in EMBRACE-II guidelines. EBRT was administered with a total dose of 45 Gy/1.8 Gy per fraction. After the introduction of VMAT, a 55–60 Gy simultaneous boost (SIB, 2.2–2.4 Gy per fraction) was given to positive pelvic or para-aortal lymph nodes. EBRT was followed by IGABT in 4 fractions with 2 applications, with the planning aim to achieve a D_90%_ of 85 Gy (EQD_2_) for the CTV_HR_.

The EBRT technique was not explicitly predefined for this study to allow for a progression of treatment technology over time. EBRT treatment evolved over time from 3D-CRT, via single plan VMAT to an ART protocol based on a library approach (plan of the day = PotD), encompassing VMAT plans for empty and full bladder plus a motion robust VMAT plan. Daily CBCT information was utilized to select the most suitable library plan [15]. A drinking protocol with the aim of comfortably full-bladder was routinely used in all groups. An empty rectum was advised, but no specific rectum protocol was provided.

### Acute toxicity scoring

Acute toxicity was evaluated by Common Terminology Criteria for Adverse Events (CTCAE) 4.03 (physician-reported; PhysRO) [30] complemented by local reporting items (daily micturition, stool consistency and stool urgency) and patient-reported (PRO) Quality of Life Questionnaire (QLQ)-C30 and EORTC QLQ-CX24 questionnaires. The evaluation was performed before treatment, weekly during treatment, and 1, 6 and 12 weeks after finishing EBRT. The patients evaluated the grade of the symptoms as none, a little, quite a bit and very much. For each PhysRO question, corresponding PRO questions were determined and analyzed.

The evaluation of stool consistency was based on Bristol stool form scale, assessing the stool type from 1–7, where 1–3 means constipation (scored separately), 4–5 normal, 6 mushy and 7 liquid stool [31]. Urine frequency 5–7 times a day was considered normal. For daily micturition the following groups were introduced: <5, 6–7, 8–14, >14 times a day. Synchronous to the introduction of VMAT, extra patient-related questions were added (QLQ 55–59) to provide more detailed information. The questions were grouped into general, GI and GU toxicity and were analyzed accordingly.

### Dose–volume parameters

The following dose–volume (DV) parameters were derived from all EBRT plans: Sigmoid V_40Gy_, Rectum V_40Gy_ and V_30Gy_, BladderV_40Gy_ and V_30Gy_ (%), Bowel V_40Gy_ and V_30Gy_ (cc), Body V_43Gy_ (cc). For all ART patients, the DV parameters for each of the three VMAT plans were weighted with the frequencies of their use in the clinic, resulting in one average weighted overall value.

### Time series

At all analyzed time points, reported side effects were corrected for their corresponding baseline values in order to avoid bias from pre-existing complaints or of symptoms that may be due to the disease itself. While the maximum EBRT dose before the start of BT can be linked with the 5 week time point, the chemotherapy (CHT) influence (e.g., nausea and diarrhea) was difficult to separate. Although almost all patients received similar chemotherapy regimens, the following PsP classification for CHT regimen was evaluated: 0 = no chemotherapy, 0.5 = capecitabine, 1 = cisplatin. An additional factor with the number of completed chemotherapy cycles was included as well.

### Statistics

All statistical calculations were performed utilizing R (www.R-project.org) [32, 33]. In case multiple parameters correlated with an effect, e.g., bladder V_30Gy_ and V_40Gy_, only the most significant univariate parameter was selected beforehand. We only selected specific parameters that seemed to be logical into the analyses. Patients with missing values for one of the side effects were excluded from the analysis only if that specific effect was under evaluation.

For relevant feature selection of DV and PsP, LASSO was used [34]. LASSO (least absolute shrinkage and selection operator) is a regression analysis method that performs both variable selection and regularization in order to enhance the prediction accuracy and interpretability of the statistical model it produces. As the number of incidences was sparse, especially in the more severe categories, higher scores were added to the lower category because glmnets’ LASSO works best for binomial parameters (glmnet is an R package that fits a generalized linear model via penalized maximum likelihood).

For the final parameters selected by LASSO, multiple logistic binomial regression was performed with only the selected parameters to maximize the information and to minimize the effect of missing values. In case a clear threshold dose was present, a Fisher’s exact test was performed. The significance of DV and PsP for the continuous variable weight loss was determined by multivariate stepwise regression at two timepoints: just before the start of BT and at the end of treatment.

## Results

From October 2014 until February 2018, 48 patients with median age of 54 (range 31–74) and FIGO stage Ib–IVb, were included in the analysis of early side effects (Fig. 1). Almost all patients received concomitant weekly cisplatin 40 mg/m^2^, 3 patients received capecitabine (Xeloda, Genentech Inc, South San Francisco, CA, USA) and four patients did not receive chemotherapy because of renal insufficiency. The number of completed cycles is shown in Fig. 1. In all, 22 patients smoked, 7 had quit and 17 patients were nonsmokers.

Three patients received 3D-CRT, 31 patients were treated with VMAT with fixed margins based on a single CT scan and 14 patients with large uterus motion received ART. A total of 18 patients received SIB-VMAT plan and 22 received extended field irradiation (with VMAT).

At the 3‑month follow-up (FU), 43 patients had complete remission, 1 patient incomplete remission, 2 patients had distant systemic progressive disease and 2 patients were lost to long-term follow-up. During further FU beyond this study (median 16 months), 2 patients developed local failure, 1 pelvic nodal failure, 2 para-aortic nodal failures and 10 patients developed distant metastases beyond para-aortic lymph nodes.

### Irradiated EBRT volumes

**Fig. 2:**
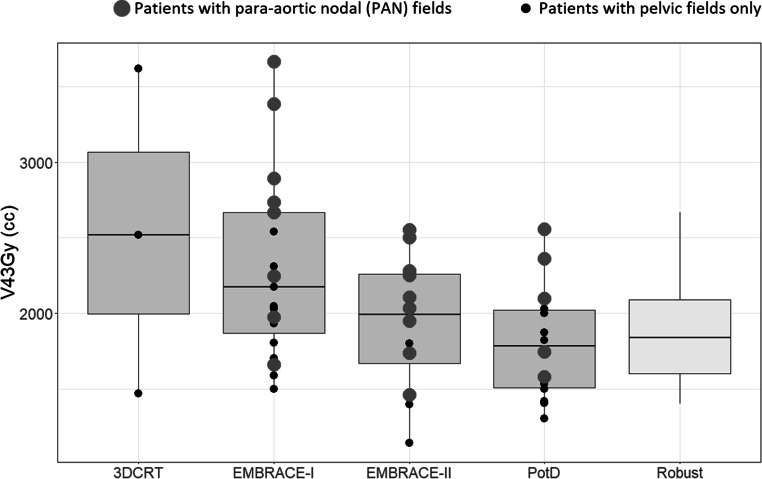
Boxplots of volumes irradiated to at least 43 Gy (V43Gy) in cc for the different irradiation techniques (EBRT treatment evolved over time from 3D conformal radiotherapy (3D-CRT), via single plan VMAT (based on EMBRACE I and later EMBRACE II guidelines) to an ART protocol based on a library approach (plan of the day = PotD)). The *Robust* equivalent is provided as a reference for the larger volume the PotD patients would have been irradiated to, in case they would not have been adapted every fraction

As shown in Fig. 2, the volumes exposed to 43 Gy (95% of prescribed dose) varied for the different EBRT techniques. V_43Gy_ was largest for the few patients receiving 3D-CRT (~2500 cm^3^), somewhat lower for patients enrolled in the EMBRACE I study (~2000 cm^3^) treated with VMAT, and lowest for patients planned according to EMBRACE II, treated with either VMAT or ART (~1800 cm^3^). In general the V43Gy for extended (PAN) fields was larger: ~2250 cm^3^ vs ~1600 cm^3^ over the entire patient group. However, for the recent techniques, the volume of the extended fields were often smaller than the small fields of the older techniques.

In only 8% of ART treatment sessions, the (larger) robust plan was chosen, while 35% of the delivered ART fractions were based on the full bladder plan and 57% for the empty bladder plan. In other words, in 92% of all ART fractions V_43Gy_ was smaller than it would have been otherwise.

**Fig. 3:**
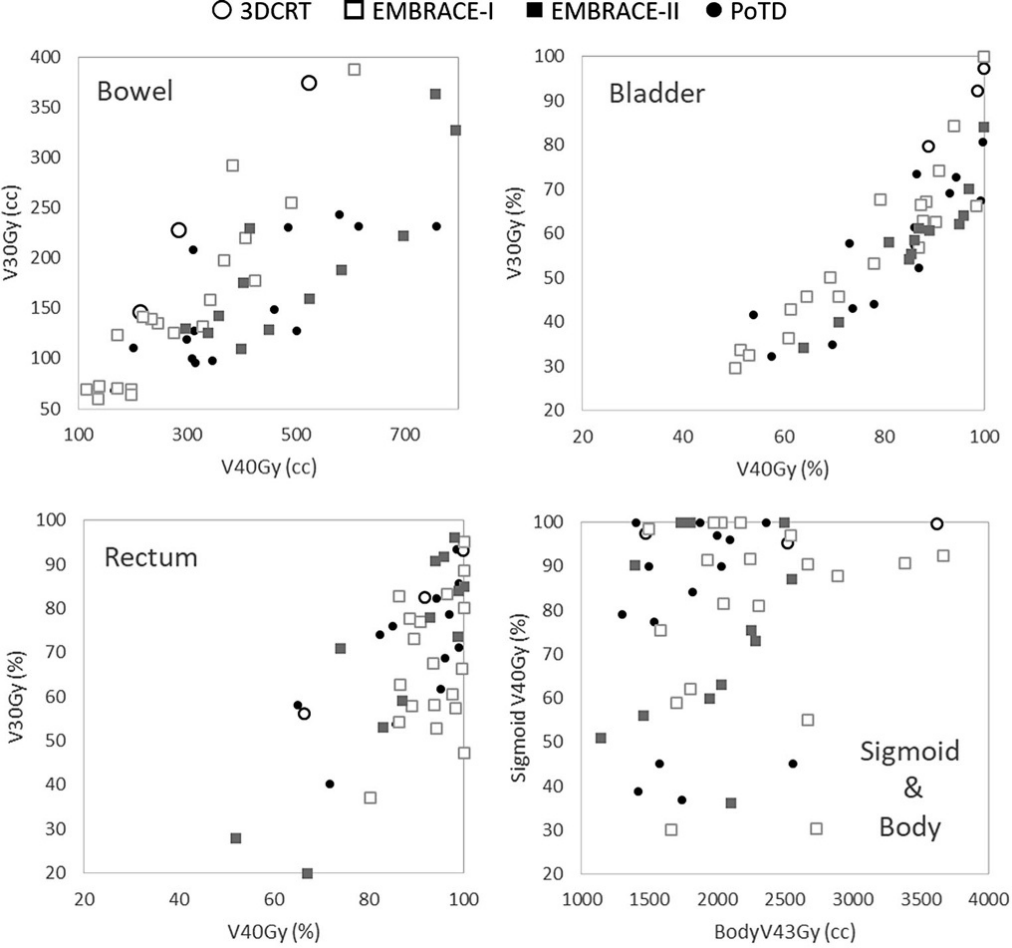
Distribution of dose–volume parameters. *PotD* plan of the day. *3DCRT* 3D conformal radiotherapy

Differences between EBRT treatment techniques and the underlying target definition protocol (EMBRACE I vs II) were larger and more significant for the higher dose–volume parameters. The volume differences between ART and EMBRACE II patients were often due to target volume variations (nodal SIB, pelvic and para-aortal lymph nodes (PLN)). In Fig. 3, the range and distribution of the various dose–volume parameters is depicted.

Table 1 summarizes the analysis of the DV parameters for primary OAR. On average, ART reduced the irradiated normal tissue volumes to 43 Gy by 80 cm^3^. More specifically, bowel tissue irradiated to ≥40 Gy was reduced by 17 cm^3^ and bladder volume irradiated receiving ≥40 Gy by 5%, respectively.

**Table 1:** Differences and significance of dose–volume parameters for the plan of the day patients compared to their robust plan

	Average Difference	*p*-value (paired t‑test)
Sigmoid V_40Gy_	−3%	0.08
Bowel V_30Gy_	−13 cc	0.2
Bowel V_40Gy_	−17 cc	**0.007**
Rectum V_30Gy_	0%	0.7
Rectum V_40Gy_	−2%	0.08
Bladder V_30Gy_	−6%	0.2
Bladder V_40Gy_	−5%	0.1
Body V_43Gy_	−80 cc	**0.0001**

### Patient and physician reported side effects

**Fig. 4:**
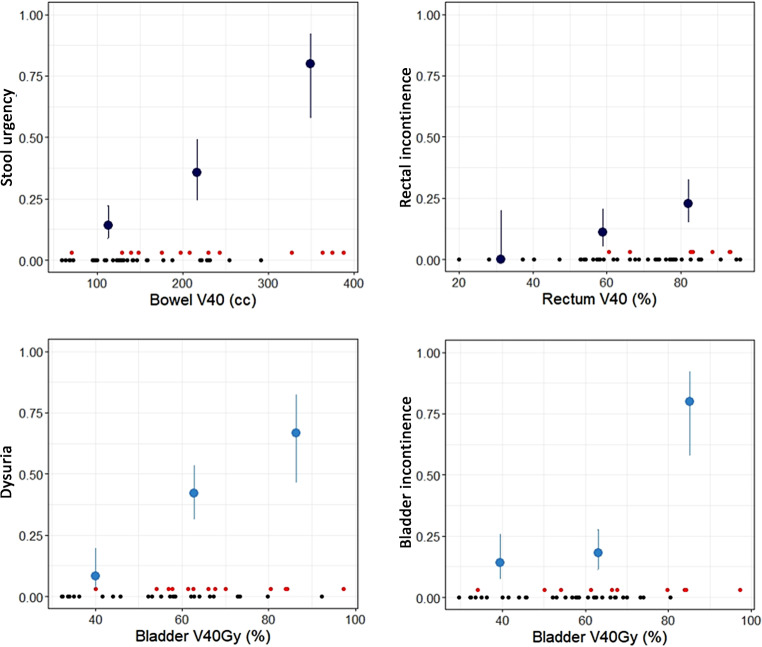
Dose–volume effect relations for CTCAE gastrointestinal (*upper graphs*) and patient reported genitourinary (*lower graphs*) parameters. The dose intervals are divided (binned) in 3 equal parts, the points are plotted at the average dose value of each bin and represent the ratio of patients with and without symptoms. The *error bars* are the 68% confidence intervals (Chi-square) and depend on the number of patients in the bin. The *black dots* are the individual patients without symptoms; *red dots* are patients with symptoms. The logistic regression uses all data at once, so the binning does not influence the significance and was done only for display purposes

The PRO evaluation was found to be more sensitive in terms of both incidence and grading of toxicity for all side effects, but most prominent for dysuria (see Appendix). Table 2 and Fig. 4 provide an overview of the side effects that were found to correlate with one or more DV parameters. In general, a worsening of symptoms during the first 5 weeks of treatment and recovery afterwards was observed. No G4–G5 toxicity was reported.

**Table 2:** Overview of dose effects that were selected by LASSO are marked with “+” and their (lowest) significance (in numbers) obtained from subsequent multivariate logistic regression analysis

Side effect	Report	Grade	Bladder V40Gy	Rectum V30Gy	Rectum V40Gy	Bowel V40Gy	Body V43Gy	Alcohol	CHT	Smoking	Age	Vomiting	Time point
*Stool urgency*	CTCAE	≥1	–	–	**+**	0.015	–	–	–	–	**+**	–	W5
	PRO^a^	≥2	–	–	–	0.02	–	–	–	**+**	**+**	–	
*Stool consistency*	–	Fluid stool	–	0.009	–	0.015	–	–	–	–	–	–	W5
*Diarrhea*	PRO	≥1	–	–	–	0.008	–	–	**+**	**+**	–	–	Fu1w
*Rectal incontinence*	CTCAE	≥1	–	–	0.006	**+**	–	–	**+**	–	–	–	W6
	PRO		–	–	0.0006	–	–	–	0.002	–	–	–	
*Rectal bleeding*^*b*^	CTCAE PRO	≥1	–	0.05	–	–	–	–	–	–	–	–	W5
*Dysuria*	PRO	≥1	0.03	–	–	–	–	–	**+**	**+**	–	–	W6
*Bladder incontinence*	CTCAE	≥1	**+**	–	–	–	–	**+**	**+**	0.0005	0.0003	–	Fu6w
	PRO^a^		0.001	–	–	–	–	**+**	**+**	**+**	0.003	–	
*Weight loss*	Measured	Continuously	–	–	–	–	0.0007	0.01	–	–	–	–	Before brachy
			–	–	–	–	0.006	0.02	–	–	–	**+**	After brachy

No significant correlation between number of chemotherapy cycles and any of the general parameters

*W5* week 5, *W6* week 6, *Fu1w* Follow-up 1 week after finishing of the treatment, *Fu6w* Follow-up 6 weeks after finishing of the treatment, *CHT* chemotherapy, *PRO* patient-reported outcome, *CTCAE* Common Terminology Criteria for Adverse Events

^a^Data available only for patients after October 2015

^b^Sparse data but clear cutoff at RectumV30Gy of 96%: below: no incidence

### Acute gastrointestinal side effects

From all gastrointestinal (GI) collected symptoms (nausea, vomiting, stool consistency, diarrhea, constipation, bloating, flatulence, stool urgency, rectal tenesmus, rectal mucus, rectal bleeding, rectal incontinence and proctitis) only stool consistency, diarrhea, stool urgency, rectal bleeding and rectal incontinence revealed dose effects in the multivariate logistic regression analysis. Stool consistency was found to be influenced by age, CHT and smoking (Table 2). The majority of patients developed diarrhea already in the first weeks of treatment, which indicates an early RT effect combined with CHT and smoking with the accumulated received dose being still small. During the treatment a slight recovery was observed (for the dynamics of side effects over time, see Appendix). Only for the patients who showed persistent diarrhea in the follow-up just after treatment was a dose effect of the Bowel parameter V_30Gy_ obtained, regardless of BT. CHT also played a role in stool urgency. Almost full recovery was present in the longer follow-up, as for the other parameters. The significant dose–volume parameter for stool urgency was the Bowel V_40Gy_, with a V_40Gy_ ~ 250 cm^3^. For rectal incontinence, a shallow dose–effect relation was observed, with very low incidence even at higher irradiated volumes; a Rectum V_40Gy_ above 80% increased the (sparse) incidence significantly in combination with CHT. Rectal bleeding seemed to be an EBRT effect as this side effect increased in week 4–5 (4 patients, 8%) and recovered quickly after treatment (6-week and 3‑month follow-up) without showing worsening after brachytherapy. Rectal bleeding was absent for Rectum V_30Gy_ < 96%, and increased to 20% for V_30Gy_ above 96%. Due to the low total number of incidences, this was borderline significant (Fisher’s exact test: *p*-value = 0.054).

### Acute genitourinary side effects

From all collected genitourinary (GU) side effects (see Appendix A), only dysuria with its highest incidence in week 5 (18 patients, 38%) and bladder incontinence at the time points week 6 and 1 week after treatment completion (14 patients, 29%) remained significant in the multivariate logistic regression. Both symptoms were influenced by CHT, smoking and age and in case of bladder incontinence, also by alcohol use (Table 2). Despite the low incidence of bladder incontinence, a clear Bladder V_40Gy_ dependency (consistent between questionnaires) was observed. For bladder incontinence, the TD_50_ of V_40Gy_ was obtained for an irradiated volume of 80–90%.

### Acute general side effects

Just before the start of BT, there was a significant correlation between weight loss and the EBRT body V43Gy (Table 2). After the first BT fraction, weight loss increased rapidly with 4 kg on average, due to limited oral intake during BT (which implies over 48 h parenteral nutrition) and the correlation of weight loss and EBRT dose became less prominent. Lumbar pain recovered during treatment compared to baseline, with the lowest incidence in week 4; afterwards it increased after epidural anesthesia and bed rest during the days of BT. The recovery of lumbar pain in the follow-up should be evaluated with caution, as some confounding factors need be taken into account such as lymphadenectomy, epidural anesthesia, bed rest during the days of BT.

## Discussion

The EBRT treatment techniques in cervix cancer radiotherapy have evolved during the decades. Several studies compared 2D, 3D and IMRT technique, providing the same local control and overall survival with significantly reduced gastrointestinal and genitourinary toxicity in IMRT treatment group [35–37].

The current pilot study was designed as a prospective observational and hypothesis generating one, with the main objective to explore correlations between acute toxicity and DVH parameters in this context. These correlations can later be used to reduce early effects as much as possible and to avoid consequential late side effects, since some acute symptoms can persist over a longer period of time and become chronic. Despite the limited number of patients and low frequency of reported acute side effects, it was possible to isolate dose-effects in GU and GI toxicity and some patient specific factors. Within this cohort there were different types of treatment planning, i.e., 3D-CRT, single plan VMAT and ART. This heterogeneity in treatment fields and nodal dose delivered, provided a larger range of dose parameters than a study of each single technique would have done.

The evolution of EBRT treatment techniques in our center over time is clearly reflected in clinical results of this study. As the variables “treatment technique” and “irradiated volume” are correlated and the subgroups were small, the clinical results obtained did not reveal a difference in toxicity between VMAT-ART and VMAT. On the other hand, there was a selection bias since VMAT-ART was intentionally limited to patients classified as “movers” with a more complicated and changing anatomy. Another contributing factor was target definition, mainly in terms of concepts (EMBRACE-II protocol). A similar trend in treated volumes was observed recently in the evolution of EMBRACE-I/-II study when evaluating treatment technique, treatment protocol and para-aortic irradiation [38].

Despite all technological advancements, accurate assessment of the actually delivered doses remains challenging. Because of daily differences in OARs volumes, DVHs of treatment plans are not representative for the whole treatment. Therefore, the analysis of the V_43Gy_ is probably a better means to assess treatment plans for EBRT of cervix cancer, as OARs move to different dose regions. In the current study V_43Gy_ correlated linearly with weight loss during the treatment.

EBRT of cervix cancer with its rather challenging target volume, surrounded by critical OARs that have a direct impact on QoL, is one of the main clinical sites where ART is explored. However, there is only a limited number of studies regarding prospective assessment of physician and patient reported outcome of acute morbidity in this context [14, 39]. Although toxicity was evaluated with standardized questionnaires, a considerable discordance was found for patient reported vs physician reported outcome, mainly in dysuria evaluation. This should be taken into account because side effects evaluated by physicians as low grade, could have an important impact on QoL [40].

When assessing patient reported outcomes, it is of utmost importance to perform a baseline assessment as many patients report GI, GU or general symptoms before treatment (Appendix, Table B.1; [8]). The incidence of GI symptoms was already two times higher in the second week of treatment, which can be explained as a direct effect of small intestines exposure, changes in irrigation, epithelial atrophy and chemotherapy [41–43], and not just by the irradiated volume. The incidence peak was in week 5–6 for the majority of the symptoms; however almost complete recovery was present at the 3 month follow-up. These findings concur with data published by Heijkoop et al. [14].

From the general symptoms, lumbar pain seems to have a dose effect in multivariate analysis, but some confounding factors need to be taken into account. For example, if patients report lumbar pain at the baseline, it could be a result of lymphadenectomy or the tumor itself.

The BT as confounding factor in DVH evaluation of all GI, GU and general symptoms was not taken into account. Most of significant parameters were found at timepoints before BT. For the parameters assessed at a later timepoint (i.e., Bladder incontinence at the 6‑week follow-up) the EBRT dose effect was highly significant even without considering BT dose.

The influence of the chemotherapy regime is difficult to assess as almost all patients got cisplatin in this study. Both the PsP parameter CHT or the number of completed chemotherapy cycles seem to influence the severity of side effects a little, but did not remain significant in the multivariate analysis (except for rectal incontinence). Prior studies reported on acute side effects with and without chemotherapy. Morbidity was described as elevated [44] or similar [45] in the combination treatment.

For patients undergoing cervix cancer treatments involving radiation therapy, numerous other studies related to QLQ evaluation were published [8, 18, 20–22]. The most reported GU symptoms were urinary frequency, cystitis and incontinence [9]; the most reported GI symptoms were diarrhea, stool urgency and rectal incontinence [10]. The majority of patients treated in those studies received 3D-CRT, i.e. 74% in [8], 63% in [10] and [9]. In an IMRT study comprising 50 patients treated with 45–50 Gy diarrhea was the most common symptom and the cut-off point for small bowel ≥G2 toxicity was V_45Gy_ > 150 cm^3^ (65% vs. 33% in V_45Gy_ < 150 cm^3^) [46]. Although toxicity was evaluated by RTOG scale, the results concur with QUANTEC constraints V_45Gy_ < 195 cm^3^ [47].

In our cohort, where 94% received VMAT and 31% VMAT-ART, low grade diarrhea, stool urgency, rectal incontinence, bleeding from GI and dysuria and bladder incontinence from GU symptoms were the most common toxicities. Only stool urgency, rectal and bladder incontinence and weight loss showed a significantly increasing distribution of incidences over the entire dose range (Fig. 3) Furthermore, a correlation between dysuria with Bladder V_40Gy_, stool urgency and incontinence with Bowel and Rectum V_40Gy_ was observed. Based on these data, the following treatment planning objectives are recommended to minimize stool urgency, rectal and urinary incontinence: bowel V_40Gy_ ≤ 250 cm^3^, rectum V_40Gy_ ≤ 80% and bladder V_40Gy_ ≤ 80–90%, respectively. These results concur reasonably with planning aims in EMBRACE-II [29], which were derived from a cohort of representative dose distributions.

With ART, less body volume was irradiated to mid and low dose volume levels than with the corresponding robust plan. It can be speculated that this could contribute to morbidity reduction. However, para-aortic extended fields may have a larger impact on normal tissue exposure than the additional organ sparing through ART. Therefore, a future comparison of adaptive and nonadaptive techniques should include a correction for extended field irradiation.

The ongoing EMBRACE-II study aims to further reduce morbidity by reducing the irradiated volume through reduced PTV margins and IMRT/VMAT following dose volume constraints for OARs. The EMBRACE-II study with a considerable patient cohort can certainly contribute to answer this clinical and scientific question and provide correlations with late morbidity, including a novel approach to distinguish between late transient and long-lasting side effects [48].

## Conclusion

This prospective study demonstrated the impact of treatment technique on quality of life (QoL) of cervix cancer patients undergoing radiochemotherapy. In general, a peak of impaired QoL was observed in treatment weeks 5–6, although this was transient with a recovery at the 3‑month follow-up. The following external beam radiotherapy (EBRT) planning objectives may be used to reduce early morbidity: bowel V_40Gy_ < 250 cm^3^ for stool urgency, rectum V_40Gy_ < 80% for rectal incontinence and bladder V_40Gy_ < 80–90% for urinary incontinence.

## Figures and Tables

**Fig. A.1:**
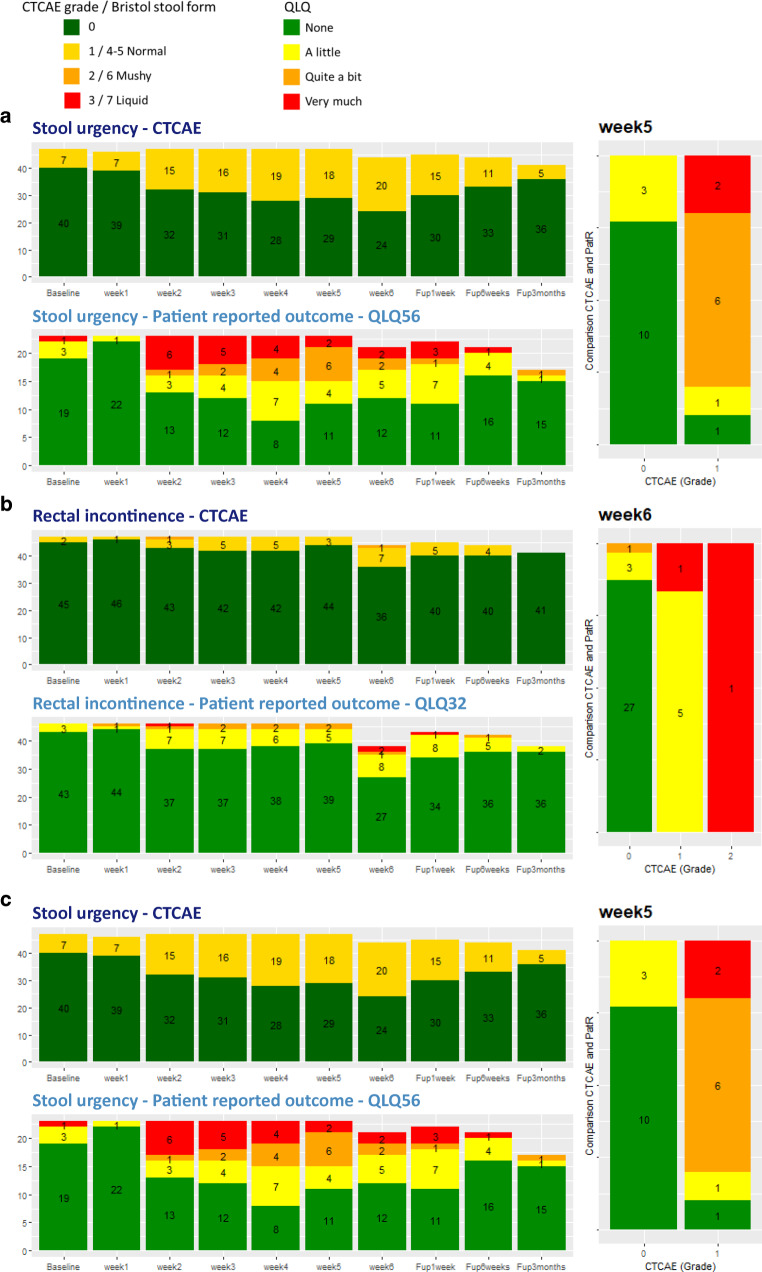
Dynamics of side effects

**Fig. A.1:**
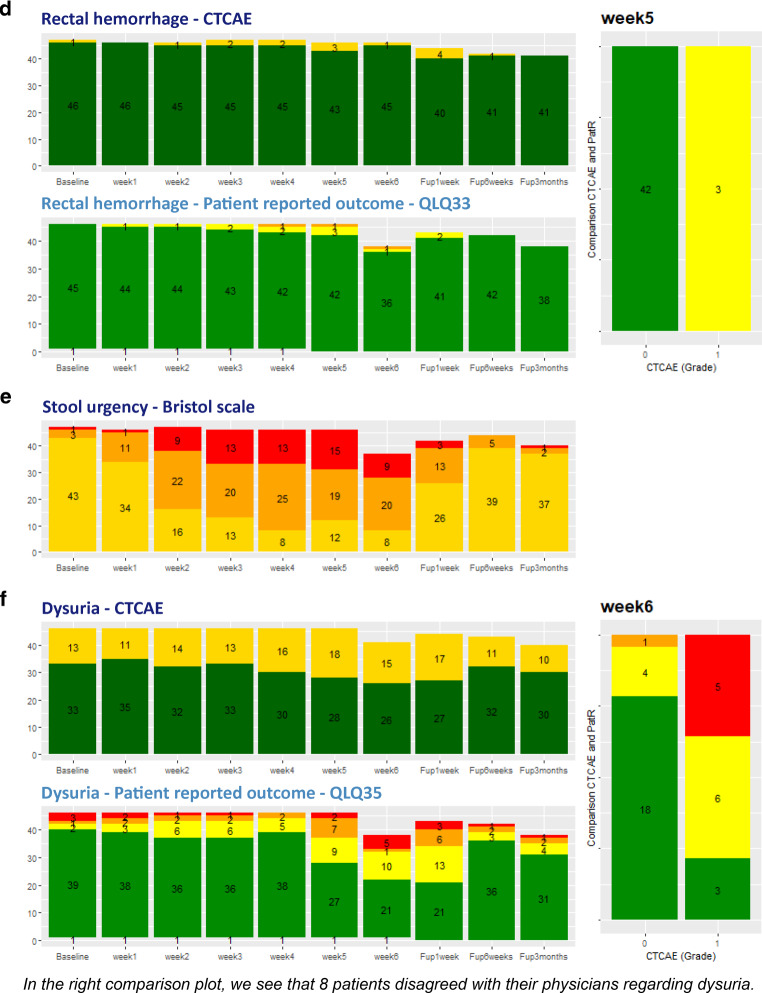
(Continued)

**Fig. A.1:**
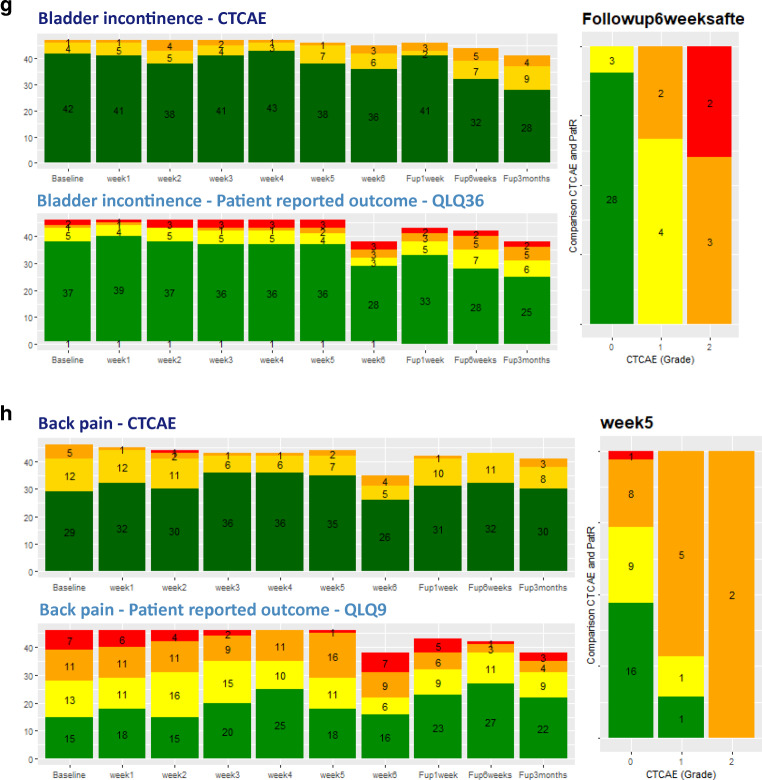
(Continued)

## Appendix A

### Collected and evaluated side effects

**GI: **stool consistency, diarrhea, constipation, bloating, flatulence, stool urgency, rectal tenesmus, rectal mucus, rectal hemorrhage, rectal Incontinence, proctitis.

**GU: **micturition during the day, nocturia, urinary urgency, bladder tenesmus, bladder bleeding, dysuria, bladder incontinence and cystitis.

**General side effects:** weight loss, fatigue, insomnia, limb edema, tinnitus, paresthesia, back pain, pelvic pain.

## Appendix B

**Table B.1:** Side effects (>grade 1) present *before* start of treatment

None or few	10–50% of the patients	>50% of the patients
Edema limbs	Pelvic pain	Fatigue
Tinnitus	Constipation	Insomnia
Paresthesia	Micturition daytime	Back pain
Nausea	Micturition nighttime	Flatulence
Vomiting	Dysuria	Vaginal discharge
Stool consistency	Bladder incontinence	
Diarrhea	Vaginal pain	
Stool urgency	Vaginal hemorrhage	
Rectal tenesmus	Vaginal inflammation	
Rectal mucus	Bloating	
Rectal hemorrhage		
Rectal incontinence		
Proctitis		
Urinary urgency		
Bladder spasm/tenesmus		
Bladder hemorrhage		
Cystitis		
Vaginal mucositis		
